# Epidemiological Transition and Strategies for the Control of Hepatitis A in Serbia

**DOI:** 10.3390/v15030753

**Published:** 2023-03-15

**Authors:** Snežana Medić, Cleo Anastassopoulou, Tatjana Pustahija, Vladimir Petrović, Nataša Dragnić, Fotini Boufidou, Athanasios Tsakris, Vladan Šaponjić

**Affiliations:** 1 Department of Epidemiology, Faculty of Medicine, University of Novi Sad, Hajduk Veljkova 3, 21000 Novi Sad, Serbia; 2 Center for Disease Control and Prevention, Institute of Public Health of Vojvodina, Futoška 121, 21000 Novi Sad, Serbia; 3 Department of Microbiology, Medical School, National and Kapodistrian University of Athens, 11527 Athens, Greece; 4 Department of Social Medicine and Health Statistics with Informatics, Faculty of Medicine, University of Novi Sad, Hajduk Veljkova 3, 21000 Novi Sad, Serbia; 5 Center for Informatics and Biostatistics, Institute of Public Health of Vojvodina, Futoška 121, 21000 Novi Sad, Serbia; 6 Neurochemistry and Biological Markers Unit, 1st Department of Neurology, Eginition Hospital, School of Medicine, National and Kapodistrian University of Athens, 11528 Athens, Greece; 7 Institute of Public Health of Serbia, “Dr Milan Jovanović Batut”, Belgrade, Dr Subotića 5, 11000 Belgrade, Serbia

**Keywords:** HAV, hepatitis A, epidemiology, outbreaks, public health, vaccine

## Abstract

Background: Improvements in socioeconomic and hygienic conditions during the past decades led to declining hepatitis A (HA) seroprevalence in many countries. Aiming at informing HA vaccination policy, we assessed current epidemiological trends in Serbia by analyzing surveillance data for 2002–2021. Methods: Data on cases and outbreaks were obtained from the Serbian national surveillance database and descriptively analyzed. HA incidence was calculated in relation to time, patients’ residence, and demographics. Results: Overall, 13,679 HA cases and 419 outbreaks were recorded with the highest incidence in the southeast. Downward HA trends were observed, while infant mortality was halved, and gross domestic product based on purchasing power parity (GDP PP) per capita, tripled. The average incidence dropped from 14.8 (95% CI 14.4–15.2)/100,000) in 2002–2006 to 1 (95% CI 0.9–1.1)/100,000)/100,000 in 2017–2021, while the number of outbreaks decreased (from 174 to 14). Sporadic cases and family clusters living in poor sanitary conditions occurred in recent years. The contact route of transmission was dominant (410/419, 97.9%). The highest average age-specific HA incidence shifted from 5–9 years in 2002–2006 to 10–19 years in 2017–2021.Serbia is transitioning towards very low HA endemicity. Enhanced surveillance and vaccination of high-risk groups are recommended as future public health priorities.

## 1. Introduction

Hepatitis A (HA) is a vaccine-preventable, acute infectious disease caused by the hepatitis A virus (HAV) [1]. HAV transmission occurs via the fecal–oral route, commonly by person-to-person contact, or by consumption of contaminated food or water. The clinical manifestation of HA correlates with age: Adolescents and adults usually have symptomatic disease in contrast to young children in whom infection is usually asymptomatic [2,3,4]. Severe forms and related complications are more frequent in older adults, immunocompromised patients, patients with chronic liver disease, and pregnant women [3]. The estimated case fatality ratio (CFR) for hospitalized patients ranges from 0.23% in adults aged < 30 years to 2.1% in those aged ≥ 49 years [3,5].

Global levels of HAV endemicity vary geographically; high levels are found in low-income areas and low or very low levels are encountered in high-income countries, resulting in large cohorts of susceptible adults prone to severe disease and complications, leading to increased disease burden [3]. In high endemicity regions, HA commonly occurs in young children < 5 years typically without symptoms. On the contrary, in high-income countries, the disease occurs predominantly among adults in high-risk groups, such as people who inject drugs (PID), men who have sex with men (MSM), the homeless, and travelers to endemic areas [3]. In middle-income countries and regions where sanitary conditions vary, an increasing percentage of adolescents and young adults are susceptible to HAV, which, in the case of infection, exposes them to the risk of more severe forms of the disease.

The epidemiology of HA is dynamic and changes with improvements in socioeconomic status, health care, and sanitation [6,7]. In addition to improved hygiene and the availability of safe drinking water, vaccination is an important part of a comprehensive plan for the prevention and control of HA. The World Health Organization (WHO) recommends universal immunization of children (≥12 months of age) on the basis of (i) an increasing trend of HA over time, including the occurrence of severe disease in older children, adolescents, or adults; (ii) changes in endemicity from high to intermediate; and (iii) having evaluated cost-effectiveness [3]. Targeted vaccination of high-risk groups should be considered in countries with low and very low endemicity. To tailor a specific vaccination strategy, countries should first estimate their national burden of HA. In addition to the use of serosurveys that assess the seroprevalence of anti-HAV IgG antibodies, decision-making requires additional studies based on surveillance data to estimate HA incidence and associated morbidity [8].

Serosurveys conducted in the early 2000s showed significant geographical differences in the level of HA endemicity in Europe with a substantial proportion of adults susceptible to HAV infection [9]. Apart from Romania, which had an intermediate level, other European countries had a low level of HAV transmission and the highest incidence of HA among older risk groups [10,11,12]. In 2021, the European Union (EU) reporting rate of HA was 0.9 cases per 100,000 population, with most cases registered in eastern Europe [13].

The Republic of Serbia is an upper–middle-income country located in central–south–eastern Europe. A previously published seroprevalence study provided evidence that HAV circulation has declined over the past four decades; thus, Serbia ranks as a very low-endemicity country nowadays, supporting the national policy of vaccination of high-risk groups [14]. National legislation opted for recommended vaccination against HA for vulnerable population groups (PID, liver transplant patients, people who work in poor hygienic conditions, and MSM) as well as for nonimmune individuals without any special risk. However, these legislative provisions have not been implemented; vaccination is currently available for international travelers only [15,16]. Population susceptibility likely increased, which is why ongoing assessment of the hepatitis A burden was deemed necessary [17].

The aim of this study was to examine the available HA surveillance data from Serbia over the period 2002–2021 in order to identify epidemiological trends and analyze possible contributing factors with the ultimate goal of updating public health recommendations, including optimizing the current vaccination strategy.

## 2. Materials and Methods

### 2.1. Study Area and Population Characteristics

Serbia is divided into five statistical regions. Since there are no published population data for Autonomous Province of Kosovo and Metohija, we did not include it in the analysis. All references to Serbia in this paper thus imply only four statistical regions (Vojvodina, Belgrade, Šumadija and western Serbia, southern, and eastern Serbia). The country is further divided into 25 districts and 29 cities, of which, 6158 settlements are the smallest administrative units [18]. During the observation period (2002–2021), the total population of Serbia decreased by 8.9% from 7,498,001 inhabitants in 2002 to 6,834,326 inhabitants in 2021. Depopulation was even more pronounced in Vojvodina where the number of inhabitants decreased by 10.3% (from 2,034,851 in 2002 to 1,825,982 in 2021). The mean age of the Serbian population increased from 40.2 (2002) and 42.1 (2011) to 43.5 years (2021), while sex distribution remained constant over the 20-year observation period (51.3% females/48.7% males). The estimated average age of the population of Vojvodina in 2021 was 43.1 years, and the proportion of male residents was 45.2% [19]. The population distribution is not homogeneous since 61.4% of the Serbian population lives in urban areas. Serbia has large regional disparities in GDP per capita, and the two regions located in the north of the country (Belgrade and Vojvodina) provide more than two-thirds of the country’s GDP (42% and 27%, respectively), which indicates the existence of a significant gap in development between northern and southern Serbia. The other two regions of central Serbia contribute to the second third of Serbia’s GDP. Given that the four regions have roughly similar population sizes, the resulting gap in regional GDP per capita is considerable. These differences in economic development are accompanied by large differences in other areas of social development, such as employment, level of education, availability of health care, and other services [20]. A recent survey indicated a regional gap in sanitation with a higher percentage of households having access to basic sanitary services in the Belgrade region and Vojvodina (99.5 and 99.6%, respectively) compared to the other two regions, Šumadija and western Serbia and southeastern Serbia (97.3 and 97.2%, respectively) [21].

### 2.2. Surveillance of HA in Serbia

In Serbia, the reporting of HA has been mandatory since 1978 [22]. Laboratory-negative serum markers of hepatitis B and hepatitis C in clinically compatible cases were used for investigation and notification of HA cases until 2007. Laboratory confirmation through HAV-specific positive immunoglobulin (IgM and IgG) antibodies in paired sera samples were increasingly used thereafter. Laboratory testing was performed in all sporadic cases and at least 10–20% of outbreak cases, while other cases were registered as epidemiologically linked. An imported case of HA is considered to be one with a positive history of traveling outside of Serbia during the incubation period (15–50 days before the onset of symptoms) with no link to a confirmed autochthonous case.

EU case definition criteria have been officially applied for the final case classification starting from 2017 [23,24]; since then, all registered cases are classified as laboratory-confirmed or probable cases (i.e., clinically compatible with epidemiological link to confirmed case(s)/source of infection). In 2017, the notification form was revised to be more comprehensive and to include additional relevant information for case classification.

Cases and outbreaks of HA are reported by physicians to the district, provincial, and national public health institutes (IPH, IPHV, and IPHS, respectively). The notification of HA is case-based, and the reporting form includes information on demographics, occupation, date of symptom onset, type and date of positive laboratory diagnostic test, case registration date, hospitalization status, vaccination status, and disease outcome. Two or more epidemiologically linked cases of HA are considered an outbreak. The following data are collected in case of an outbreak: year and place of occurrence, number of exposed, infected, and hospitalized people, including their residence, age, and sex, suspected source of infection, and most likely mode of transmission, as well as adopted control measures. Data on HA cases and outbreaks are reviewed and entered into regional, provincial, and national surveillance electronic databases.

Upon receipt of the notification form, IPH epidemiologists undertake an epidemiological investigation of each case. Additional data, such as list of symptoms, food, and water consumption details, recent history of travel abroad, other risk exposures during the maximum incubation period, and data on epidemiologically linked cases, are obtained through patient interviews and entered into epidemiological questionnaires. The epidemiologist informs the authorities and the public in the event of an outbreak and proposes the implementation of appropriate disease control measures.

### 2.3. Study Setting, Data Sources and Analysis

A retrospective, population-level study was performed using surveillance data for HA in Serbia from 2002 to 2021. Data on HA cases and outbreaks were obtained from the Serbian national IPHS surveillance database, which is comprehensive of the entire population. Codes B.15-B.15.9 of the International Statistical Classification of Diseases and Related Health Problems, Tenth Revision (ICD-10) were used to search the database [25]. Cases living outside Serbia were excluded. Outbreak reports were also collected and analyzed. Detailed information on HA cases (hospitalization status, number of confirmed and probable cases, exposure and risks, route of HAV transmission, and characteristics of cases, including vaccination history) was only available for Vojvodina through the IPHV surveillance database.

The descriptive statistics for all variables related to HAV infection were provided in terms of number of cases (n), proportions (%), and annual crude incidence of HA per 100,000 inhabitants. The average incidence was estimated with a 95% confidence interval (95% CI). Age-specific incidence was calculated per 100,000 population in each age group (<5, 5–9, 10–14, 15–19, 20–29, 30–39, 40–49, 50–59, and ≥60 years). Mortality per 100,000 inhabitants was calculated. Official population estimates for Vojvodina and central Serbia for the corresponding age categories were used as numerators [19]. Notification times were grouped into four five-year subperiods. Hospitalization rates were calculated. The chronological, topographic, and descriptive analysis of study outcomes was carried out at IPHV. We used Quantum GIS (QGIS), version 3.4. for map creation.

### 2.4. Socioeconomic, National Health Indicators, and HAV Incidence

We analyzed socioeconomic and health indicators in relation to the crude incidence of HA in Vojvodina and Serbia per 100,000 individuals. The gross domestic product per capita based on purchasing power parity (GDP PP) in U.S. dollars represented an economic indicator reflective of the standard of living [26]. The infant mortality rate (IMR), defined as the number of infant deaths before 1 year of age per 1000 live births in a given year, was used as an indicator of the quality of national healthcare [27,28]. Spearman’s correlation analysis was used to test the possible correlation between examined variables (GDP PP, IMR, and incidence of HA).

### 2.5. Ethics Statement

Notification data were collected in the frame of national surveillance as required by law [29]. Patient identification data were coded, merged, and entered into a specially designed Excel database that was used for analysis. Access to data was provided exclusively to authorized IPHV researchers. Ethical approval for the study was obtained from the Ethics Committee of IPHV (Number: 01-1630/1-1, 2 December 2022).

## 3. Results

### 3.1. Hepatitis A Incidence Trends and Characteristics of Cases

**Figure 1:**
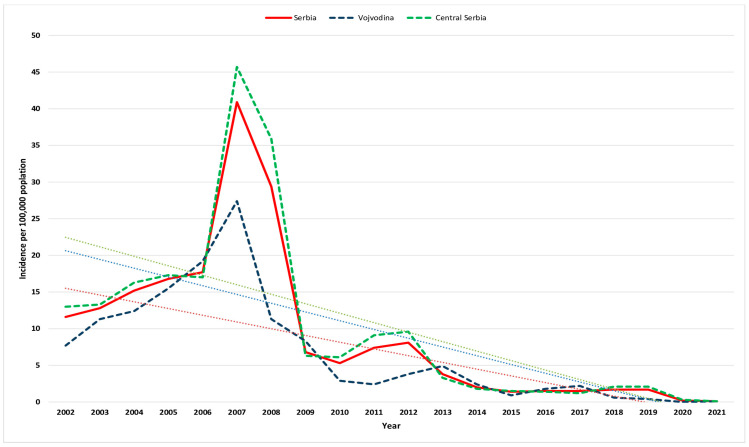
Crude incidence of hepatitis A per 100,000 population in Vojvodina, central Serbia, and Serbia (blue, green, and red lines, respectively) from 2002 to 2021. The corresponding trendlines are also shown.

A total of 13,679 HA cases were reported in Serbia in the period 2002–2021 (Table 1). The average incidence rate for the entire 20-year period was 9.5 (95% CI 9.3–9.6)/100,000 ranging from 0.1/100,000 in 2021 to 40.9/100,000 in 2007 (Figure 1). The average incidence dropped from 14.8 (95% CI 14.4–15.2)/100,000 in 2002–2006 to 1 (95% CI 0.9–1.1)/100,000 in 2017–2021. Most cases (n = 6596; 48.2%) were observed in the period 2007–2011 when the average HA incidence peaked at 18.0 (95% CI 17.6–18.5)/100,000. The overall male-to-female ratio was 1.3:1. The average specific incidence was in favor of men throughout the study period [11.0 (95% CI 10.8–11.3)/100,000 in males vs. 8.0 (95% CI 7.8–8.2)/100,000 in females], but male predominance decreased over the years. In the period 2017–2021, male predominance was evident in the 20–49 age group (Table S1). Overall, five deaths related to HA were recorded and thus overall mortality was 0.06/100,000 population. All deaths were registered in the period 2002–2008.

Downward trends characterized HA incidence from 11.6/100,000 population (867 cases) in 2002 to 0.1/100,000 population (8 cases) in 2021 (Figure 1). Similar incidence rates of HA were observed between the two regions except in 2007–2008 when central Serbia presented a substantially higher incidence rate of HA.

**Figure 2:**
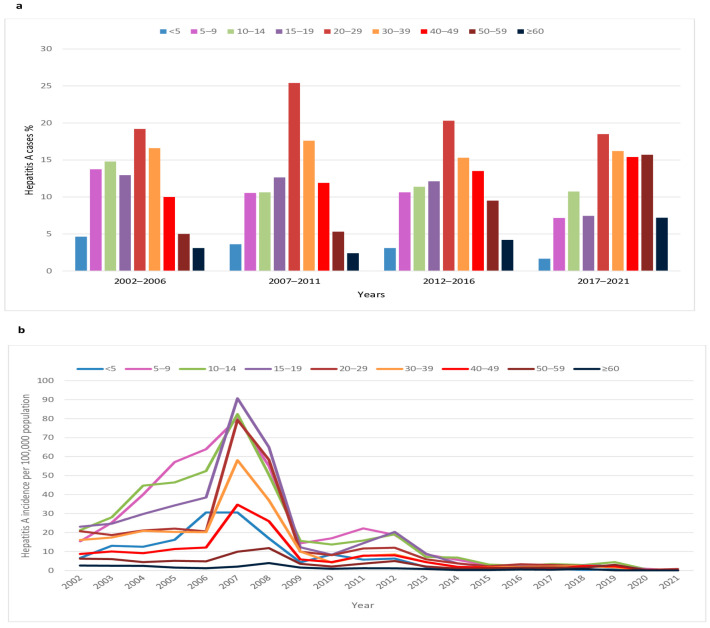
Age-specific hepatitis A (**a**) cases and (**b**) incidence per 100,000 population in Serbia, 2002–2021.

In the period 2002–2021, the highest percentage (18.5–25.4%) of cases was recorded among younger adults aged 20–29 years (Figure 2a). The share of adults aged 30–39 years in the total number of patients remained constant (15.3–17.6%) throughout the study period. The share of cases among adults aged 40–49, 50–59, and ≥60 years increased from 10.0%, 5.0%, and 3.1% in the period 2002–2006 to 15.4%, 15.7%, and 7.2%, respectively, during the last five years (2017–2021). The percentage of children aged < 5, 5–9, 10–14, and 15–19 years decreased from 4.6%, 13.7%, 14.8%, and 12.9% in the period 2002–2006 to 1.7%, 7.2%, 10.7%, and 7.4%, respectively, in the period 2017–2021.

In the period 2002–2006, the highest average incidence rates of HA were observed in children aged 5–9 and 10–14 years (39.7 and 38.4/100,000); these are almost 3 times higher than the rates observed in children aged < 5 years (Figure 2b). A shift in the age-specific incidence towards adolescents aged 15–19 years (average incidence 39/100,000) was observed in the period 2007–2011. During the past decade, the incidence of HA decreased and stabilized at low levels in 2020–21 (range 0–0.9/100,000). The highest rates were found in older children and adolescents (range 0–20.3/100,000). The lowest age-specific incidence of HA was observed in older adults ≥ 60 years during the entire 20-year period (range 0.05–3.9/100,000).

**Figure 3:**
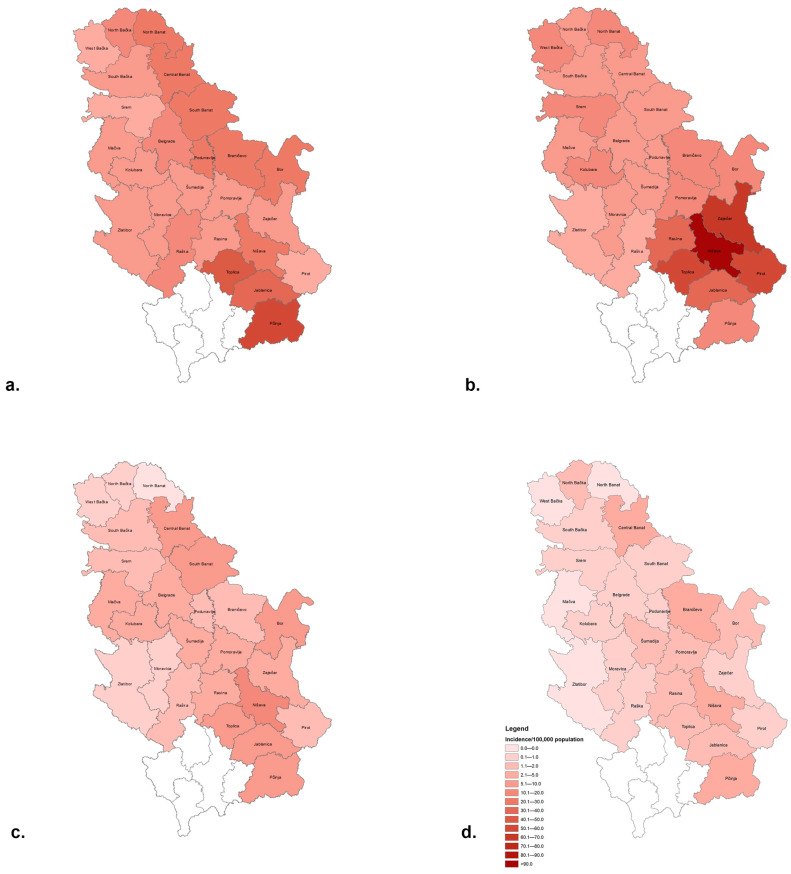
Average district-level incidence rates of hepatitis A in the subperiods (**a**) 2002–2006, (**b**) 2007–2011, (**c**) 2012–2016, and (**d**) 2017–2021.

The average district-level incidence rates of HA over the 20-year period divided into 5-year subperiods are shown in Figure 3. Compared to central Serbia where the average HA incidences in the subperiods 2002–2006, 2007–2011, 2012–2016, and 2017–2021 ranged from 3.9–51.9, 4.1–106.2, 0.4–12.5, and 0.1–2.5/100,000 inhabitants, the rates notified in the northern province of Vojvodina were slightly lower (2.5–26.7, 6.7–15.3, 0.1–8.6, and 0–1.7/100,000 inhabitants). In the first decade of the study period, the average incidence of HA ranged between 2.5–106.2/100,000 inhabitants and was highest in southern–eastern Serbia. Thus, in the period 2002–2006 (Figure 3a), the highest incidence rates were registered in the districts of Toplica and Pčinja (up to 51.9/100,000 in the latter district). In the period 2007–2011 (Figure 3b), the highest incidence was recorded in Pirot, Toplica, Zaječar, and Nišava districts (up to 106.2/100,000 in the latter). The high incidence of HA in the Nišava region is the result of a large outbreak that occurred in 2007 in that area (716 cases were recorded in October–November 2007). The average incidence of HA in the subperiods 2012–2016 and 2017–2021 declined sharply (0–12.5/100,000) but still remained highest in Nišava in both sub-periods (12.5 and 2.5/100,000, respectively) (Figure 3c,d).

The seasonal distribution of HA cases is depicted in Figure S1. The lowest percentage of HA cases was notified in spring/early summer (3% in May and 3.1% in June) and the highest between October and December (45% overall).

### 3.2. Outbreaks of Hepatitis A in Serbia

A total of 419 outbreaks with 2990 cases were reported in Serbia in the observed period, which accounts for about one-fifth (21.9%) of all hepatitis A patients (Table 2). Outbreaks were five times less frequent (n = 70; 16.7%) in the period 2012–2021 compared to 2002–2011 (n = 349; 83.3%) when most outbreaks occurred. Cases of HA in the past decade occurred sporadically and in the form of small family outbreaks in communities with poor sanitary conditions. The close contact, i.e., person-to-person, route of transmission was established in 410 out of 419 outbreaks (97.9%) that affected 2927 (97.9%) of all affected patients during outbreaks. Outbreaks of foodborne (n = 2; 0.5%) or waterborne (n = 7; 1.7%) origin were rare and affected only 63 patients (2.2%); no such outbreaks were observed in the last ten years of the observed period. For the majority of cases and in all notification periods, the source of exposure was reported to be unknown or was not specified. In community-wide outbreaks that spread by person-to-person route of transmission, “contact with an infected person” was the most frequent exposure risk.

### 3.3. Characteristics of Hepatitis A cases and Outbreaks in Vojvodina

A total of 2696 HA cases and 79 outbreaks were notified in Vojvodina (19.7% and 18.9% of all HA cases and outbreaks recorded in Serbia, respectively) (Tables S2 and S3). Of the 2696 HA cases, nearly half (n = 1279; 47.4%) were hospitalized (Table S2). On average, 64 patients (range: 0–336 patients) were hospitalized annually due to HA. Hospitalization rates decreased from 51.2% in the period 2002–2006 to 30.6% in 2017–2021. In the period 2002–2016, the hospitalization rate due to HA ranged from 10.5–78.7% with two prominent peaks in the epidemic years 2006–2007 and 2012–2013 (61.5–74.5% and 65.7–78.7%, respectively). The age structure of hospitalized cases corresponds to the age structure of patients in a given year. In the period 2002–2011, children and adolescents aged ≤ 19 years and adults aged ≥ 20 years were almost equally hospitalized. The percentage of adults among hospitalized patients increased from 51.3% (n = 579/1128) in the period 2002–2011 to 78.1% (n = 118/151) in the period 2012–2021.

The majority of HA cases in Vojvodina were recorded in outbreaks (1662/2696; 61.6%), but the proportion of outbreak cases decreased 2.5 times: from 67.7% in the period 2002–2006 to 27.4% in the period 2017–2021 (Table S2). Among outbreak cases, 332 (19.9%) were laboratory-confirmed, 428 (25.8%) were probable, and nearly half of the cases (all recorded in the period 2002–2006) remained unclassified (n = 902; 54.3%). A record number of outbreaks (19 with a total of 317 patients) was recorded in 2007. The number of cases per outbreak decreased 4-fold from an average of 33 in the period 2002–2006 to 8 in the period 2012–2016. After 2012, massive outbreaks in schools and Roma settlements were replaced with small-scale clusters, mostly in families with poor hygiene. In 2017, only one outbreak was reported with 17 cases, and there were no registered outbreaks of HA in the period 2018–2021. Close, i.e., person-to-person, contact was the most probable route of transmission in all recorded outbreaks. Accordingly, “contact with an infected person” was the most frequently reported exposure risk among outbreak cases (n = 1247; 75%), while for the remaining cases, the risk was “unknown” or not specified. Most outbreaks occurred in the general population (n = 55, 69.6%), while outbreaks in Roma communities (n = 9; 11.4%), schools (n = 11; 14.0%), hospitals (n = 2; 2.5%), and immigrant camps (n = 2; 2.5%) were less frequent and occurred mainly in the period 2002–2011 (Table S3). In response to the outbreaks, measures were implemented to identify epidemiologically linked, unrecognized cases of HA, as well as to follow up on at-risk persons who were in contact with patients; potentially exposed individuals were medically supervised until the end of the maximum incubation period. Recommendations were given for improving living conditions and general and personal hygiene in communities, schools, and camps with a special emphasis on improving hand hygiene. Reactive immunization strategies were not implemented.

Apart from outbreaks, sporadic cases of HA were also registered in Vojvodina occasionally. Data on patients’ mobility within the maximum incubation period were available for 295/325 (90.8%) patients in the period 2012–2021. Data on travel outside the country were positive for 21 patients (7.1%), and data on travel to another place within the country were indicated for 39 (13.2%) patients, while the majority of patients stated that they did not leave their residence (n = 235, 72.3%). Most of the travel-related cases (n = 14, 66.7%) visited countries of the former Yugoslavia. There were no reported intravenous drug users or men who have sex with men (MSM) among the cases. None of the patients had been vaccinated against hepatitis A.

### 3.4. Economic, National Health Data and the Incidence of Hepatitis A in Serbia

**Figure 4:**
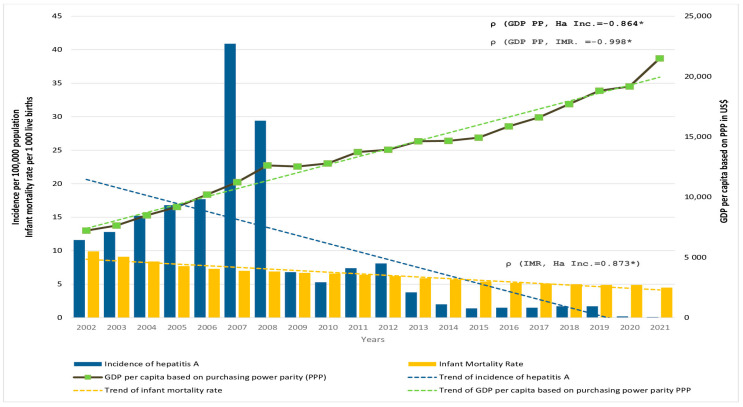
Incidence of hepatitis A (HA Inc.) per 100,000 population (blue bars, left axis), infant mortality rate (IMR) per 1000 live births (yellow bars, left axis), and GDP per capita based on purchasing power parity (GDP PPP) in US dollars (solid black line, right axis) in Serbia in the period 2002 to 2021. The corresponding trendlines are also shown. * *p* < 0.001.

Decreasing trends of HA incidence (from 11.6 notified in 2002 to 0.1 per 100,000 population in 2021) in Serbia were followed by a similar trend in IMR, which was halved from ~10 per 1000 live births in 2002 to ~4.5 per 1000 live births in 2021. In the same period, the GDP PP tripled (from $7225 in 2002 to $21,503 in 2021) (Figure 4). There was a significant positive correlation between the incidence of HA and the IMR (ρ = 0.873, *p* < 0.001), and a significant negative correlation between GDP PP and incidence of HA (ρ = −0.864, *p* < 0.001) as well as between the GDP PP and IMR (ρ = −0.998, *p* < 0.001) in Serbia.

## 4. Discussion

To the best of our knowledge, this is the first population-based study that allows for a comprehensive assessment of HA incidence trends over the past two decades in Serbia based on surveillance data. We found a decreasing trend of HA incidence with significant regional differences and the highest rates notified in the southeast of the country. The age-specific incidence of HA shifted from childhood (5–14 age group) to adolescence (15–19 years) with an increasing percentage of affected adults throughout the 20-year study period. Despite the regional disparities in development particularly in the south compared to the north of the country, the improvement of health care and economic growth in the observed period in Serbia was accompanied by a decrease in the incidence of HA. Large-scale outbreaks that occurred in the general population, schools, and Roma communities in the period 2002–2011 have been replaced by small clusters of HA in areas with poor sanitary conditions. Contact with an infected person was the most common risk reported, and none of the infected cases had been vaccinated against hepatitis A.

A serosurvey conducted on samples collected in 2015–2016 showed that HAV seroprevalence in Vojvodina increased with age from 3.1% in the age group 5–9 years to 89.6% in people ≥ 60 years old [14]. By the age of 30 years, only 5% of individuals are seropositive, leaving the majority of children and young adults susceptible to HAV. The estimated age of midpoint immunity (AMPI) progressively increased from 14 years in 1978–1979 to 55 years in 2015–2016. These changes reflect the epidemiological transition of Vojvodina and Serbia from high to very low HAV endemicity. The results of this study corroborate these findings.

The incidence of HA during the 20-year period shows a decreasing trend in Serbia similar to the European Union/European Economic Area (EU/EEA) countries. The incidence of HA in the EU/EEA decreased from 10.0 to 2.5 per 100,000 between 1997 and 2011 and from 5.1/100,000 in 2017 to 0.9/100,000 in 2021 [13,30]. HAV endemicity ranged from very low (western and northern EU countries) to intermediate (southern and eastern EU countries). International variations of the annual incidence of HA among EU member states are mainly associated with disparities in socioeconomic development, hygiene, and sanitation. In general, central and eastern EU member states, especially those neighboring Bulgaria and Romania, reported higher HA incidence rates compared to other countries [13,31,32]. In countries with very low endemicity, HA affects mainly vulnerable populations, including travelers to high endemicity areas, marginalized groups, PID, and MSM. In 2021, the EU/EEA notification rate (0.9/100,000 population) was the lowest since the beginning of HA surveillance at the EU level, mostly influenced by the COVID-19 pandemic. Less frequent international travel, lockdowns, restaurant closures, fewer gatherings, and social interactions but also insufficient reporting of infectious diseases during the pandemic contributed to these trends [13].

Higher incidence rates of HA were observed in Serbia compared to EU/EEA notification rates in the period 2002–2013; thereafter, incidence rates were lower in Serbia perhaps as a consequence of massive foodborne outbreaks that occurred in 2013–2014 and afterward, as well as in the MSM population in 2017–2018 [33]. Other reasons accounting for these differences could pertain to better accessibility to laboratory diagnostics and more comprehensive surveillance in EU/EEA member states. International disparities in the notification rates of HA must be interpreted with caution given the differences in national surveillance systems, adopted case definitions of HA, and the varying degree of underreporting at the country level. Moreover, the cyclic recurrence of HA incidence was observed in low-endemicity settings both within the general population and in high-risk groups [13].

In Serbia, the incidence of HA dropped below 2/100,000 inhabitants after 2014, and in the period 2017–2021, the incidence rate ranged between 0.1–1.7/100,000 population. Similarly, to the situation in the EU, in the last five years, including the recent pandemic years 2020–2021, the lowest incidence rates of HA (0.1–0.2/100.000) were recorded in Serbia since the beginning of surveillance of all communicable diseases. This can be explained by the reasons mentioned above but also by the severe underreporting and limited laboratory diagnosis of all infectious diseases apart from COVID-19.

Age and sex differences among the affected population are similar in Serbia and the EU/EEA. The highest HA notification rates in EU/EEA were observed in children 5–14 years followed by the 0–4- and 15–24-year age groups [13,32]. Male predominance was also observed but mostly in patients aged up to 45 years.

Substantial regional differences in incidence rates of HA were observed. Apart from the disparities in socioeconomic differences and living standards between regions and districts, there are also differences in the frequency of reporting infectious diseases among districts.

Outbreaks of HA are usually associated with poor sanitation. Outbreaks with a person-to-person route of HAV transmission are more common in communities with poor sanitation, among the homeless, the MSM population, PID, and in overcrowded communities. These factors are also associated with a higher relative risk of hospitalization [34,35]. Person-to-person transmission of HAV via the fecal–oral route was the dominant route of HAV transmission in outbreaks in Serbia. A small number of outbreaks through contaminated food and water were recorded in the period 2002–2011. Given that the source of exposure remained unknown or unspecified for most outbreak cases, there is a possibility that additional outbreaks may have taken place, but they were not recognized and investigated.

In Serbia, HA outbreaks were five times less frequent in the last decade and mostly occurred in families living in poor sanitary conditions. HAV transmission from person to person within families stands out as a likely risk factor for HAV infection [36]. Prolonged shedding of HAV before and after the onset of jaundice, combined with a lack of good hygiene practices and sharing contaminated objects in family households, favors HAV transmission from person to person. In the household, susceptibility to HAV infection is associated with younger age, while HAV immunity is significantly associated with older age [36,37]. Indoor outbreaks require rapid action measures to control the infection. The vaccination of susceptible household contacts may prevent the viral spread. With the exception of 2021, outbreaks continued to occur in Serbia, providing evidence of HAV circulation in the population but with significant regional differences. This is supported by the fact that since 2017, no HA outbreaks have been registered in Vojvodina in contrast to the rest of the country.

In the EU/EEA, several multinational outbreaks of HA have been reported in the past decade, including travel-related, community-wide, and foodborne outbreaks linked to the consumption of frozen fruits, dried vegetables, and fresh food products [38,39,40,41]. “Seeding events” when HAV was introduced into a susceptible population via a primary food- or travel-related case have led to community-wide transmission in some countries [29,42]. An unprecedentedly large and prolonged multinational outbreak of HA in the EU/EEA in 2017–2018 affected the MSM population [32,43,44]. The increase of natural immunity in the MSM population after the 2017–2018 outbreak in addition to increased awareness of HA transmission and practicing good hygiene as well as increased vaccine uptake among at-risk groups have contributed to less frequent outbreaks in this population nowadays.

Travel to endemic areas is still a major risk factor for HAV infection in the EU/EEA. The number of travel-associated HA cases is much higher in developed countries compared to Serbia, but these may be also underestimated due to underreporting [13,17]. Based on the data obtained for Vojvodina (7.1% travel-related cases in the period 2012–2021), it can be concluded that the number of these cases in Serbia is not negligible. Counseling and vaccination of travelers against HA are poorly represented (only 165 administered vaccines against HA in Serbia in 2021) [45]. Sexual orientation and practices may play an important role in HAV transmission [46]. A recent multinational outbreak of HA in the MSM population in the EU/EEA revealed the need for better recording of the MSM status of HA cases [43,44,47]. Surveillance data do not indicate the occurrence of HA outbreaks among the MSM population in Serbia. Although the proportion of MSM among patients with HA is unknown, it is realistic to assume that there were cases that did not disclose this piece of information due to fear of stigmatization. This is supported by the preponderance of men among the patients (M:F 1.3:1) over the 20-year period.

The hospitalization rate related to HA in Vojvodina was unexpectedly high (47.4%) for such a mild disease, although higher hospitalization rates (85.5%) were recently recorded [8]. Decreasing trends of hospitalization rates (from 51.2% in 2002–2006 to 30.6% in the period 2017–2021) may have been influenced by the overuse of hospitalization or much broader criteria for hospital admission in the period 2002–2011. Patients who were difficult to control, especially members of hard-to-reach communities, such as Roma or migrants, were hospitalized only to avoid further transmission of HAV in susceptible populations and to ensure appropriate follow-up. As the share of patients in those communities decreased over time, the indications for hospitalization became stricter and, nowadays, refer primarily to adult patients with serious illnesses.

As in Serbia, the seasonal distribution of HA cases in the EU/EEA countries peaks between September and November [13]. This seasonal pattern can be explained by the return of children to schools and employees to work after vacations, leading to more frequent person-to-person interactions. Return from summer trips and visits to endemic areas that increased the frequency of sexual contacts and eating habits (that may promote fecal–oral transmission) along with the long incubation of HA are possible reasons for this autumn peak [48].

National economic and health system indicators are often used to analyze possible links to disease trends in disease burden assessments and seroprevalence studies [49]. The decrease in the incidence of HA in Serbia was accompanied by a rise in GDP PP as an indicator of better living conditions and a decline in IMR as an indicator of improved health care. A better-balanced economic landscape would equalize the existing disparities between the north and south and enable social progress that would share the benefits of growth in all parts of the country. Critical factors in attaining equitable sanitation in Serbia encompass reducing geographical disparities, overcoming the barriers faced by vulnerable and marginalized groups, and addressing financial affordability issues [50].

Inactivated hepatitis A vaccines are highly immunogenic and effective at preventing HAV infections and provide strong and sustained immune responses [3,51,52]. The decline in the incidence of HA in most middle-developed and almost all developed countries has led to an increase in the population susceptible to HAV, which has not been accompanied by an increased vaccination rate [30]. Although the national immunization policy mandates the vaccination of vulnerable population groups (PID, liver transplant patients, persons working in poor hygienic conditions, and MSM) with two doses of HA vaccine and recommends the vaccination of nonimmune persons without special risk, the implementation of this legal provision has been postponed until further notice [15,16].

The surveillance of communicable diseases in Serbia has been suboptimal in the past decade mostly due to the underutilization of laboratory diagnostics. The recently established comprehensive surveillance of communicable diseases through the electronic public health service should reduce underreporting in the future [53]. The national surveillance system must be further improved through updated case definitions of infectious diseases, including classification criteria and minimum reporting data for surveillance purposes. Unifying the outbreak research methodology with the application of analytical methods, as well as improving outbreak reporting, is imperative for strengthening surveillance.

Some limitations of our study should be mentioned. HA documentation is influenced by the availability of laboratory testing. Thus, subclinical infections, mostly in children who are more likely to develop the mild disease, were conceivably not captured by the surveillance system. Substantial underreporting was observed for all infectious diseases, including HA, in the last five years, especially during the 2020–2021 pandemic period, which renders the interpretation of long-term disease trends difficult. Underreporting, along with the inevitable omission of asymptomatic cases, creates the assumption that the actual number of HA cases is higher than presented. Established disease trends are largely influenced by changes in disease reporting and notification throughout the study period. Insufficient data on exposure to HAV during the incubation period made it impossible to accurately determine the proportion of vulnerable population groups among patients, such as MSM or PID. Moreover, clinical features of HA, including the proportion of severe cases and complications as well as HA case classification at the national level, were not discussed. Information on the molecular sequencing of circulating viruses could be useful for linking cases and outbreaks, but the molecular characterization of HAV strains was poorly represented in Serbia. The impact of vaccination was not assessed since the vaccination history of most patients was negative. However, the results certainly suffice to characterize HA incidence trends in Serbia and analyze the necessary public health strategies in the future.

## 5. Conclusions

The true rate of HAV infection in a country cannot be precisely determined due to the typically asymptomatic disease course among the youngest population; however, data on symptomatic cases can still provide insight into disease trends that can be used to design public health strategies. Along with the recently obtained seroprevalence data, this study provides evidence of Serbia’s transition to very low HAV endemicity nowadays. A decreasing trend in the incidence of HA was found during the past two decades with the highest rates recorded in the southeast of the country. The increase in the proportion of adults among patients is worrisome given that the susceptible adult population is prone to more severe forms of the disease. Despite the low incidence rate of HA in recent years, HAV continues to circulate in pockets of vulnerable communities with poor sanitary conditions, especially in south–eastern Serbia. The results of this study can be a guide for designing a more effective public health policy in Serbia regarding the control of HA in the future. This policy would have to rely on improving living conditions and equalizing existing disparities between northern and southern regions to reduce the risk of HAV transmission and outbreaks in economically underdeveloped areas of the country. Measures aimed at improving hygiene and sanitation and rapid and effective response to outbreaks, including timely contact tracing and reactive immunization, could reduce the likelihood of community-wide outbreaks in the future. Priority should be given to enhancing surveillance, particularly among patients belonging to vulnerable population groups. In addition, decentralization and improved access to laboratory testing are needed, including molecular characterization of HAV, as well as international sharing of sequences to rapidly detect and diffuse multinational epidemics. Furthermore, effective collaboration between the public health and food safety sectors is important to reduce the likelihood of foodborne outbreaks. Given the continued circulation of HAV and the obvious risk of transmission to a growing cohort of susceptible adults, the urgent implementation of vaccination of high-risk groups against HA is necessary for Serbia. However, prior identification of these groups and an appropriate promotional campaign with clear messages about the safety and efficacy of vaccination are a prerequisite.

## Figures and Tables

**Table 1:** Hepatitis A notification data in Serbia, 2002–2021.

	2002–2006	2007–2011	2012–2016	2017–2021	Total
	**n (%)**	**n (%)**	**n (%)**	**n (%)**	**n (%)**
Cases	5524 (40.4)	6596 (48.2)	1196 (8.7)	363 (2.7)	13,679 (100)
Sex					
Male	2991 (54.1)	3916 (59.4)	654 (50)	193 (54.0)	7754 (56.7)
Female	2533 (45.9)	2680 (40.6)	542 (50)	170 (46.0)	5925 (43.3)
HA-related deaths					
n	2	3	0	0	5
Mortality ^1^	0.02	0.04	0	0	0.06
Average Incidence (95% CI) ^1^					
Republic of Serbia	14.8 (14.4–15.2)	18.0 (17.6–18.5)	3.4 (3.2–3.6)	1.0 (0.9–1.1)	9.5 (9.3–9.6)
Vojvodina	13.2 (12.5–13.9)	10.6 (9.9–11.2)	2.8 (2.4–3.1)	0.7 (0.5–0.8)	7.0 (6.7–7.2)
Central Serbia	15.4 (14.9–15.9)	20.7(20.2–21.2)	3.6(3.3–3.8)	1.2(1.0–1.3)	10.4(10.2–10.6)
Average Sex-specific Incidence (95% CI) ^1^					
Male	16.5 (15.9–17.1)	22 (21.3–22.7)	3.8 (3.5–4.1)	1.1 (1.0–1.3)	11.0 (10.8–11.3)
Female	13.2 (12.7–13.7)	14.3 (13.7–14.8)	3.0 (2.7–3.2)	1.0 (0.8–1.1)	8.0 (7.8–8.2)

^1^ per 100,000 population.

**Table 2:** Reported cases and outbreaks of hepatitis A in Serbia, 2002–2021.

Subperiod (Years)	Hepatitis A Outbreaks n (%)	Outbreaks per Transmission Route						Overall Hepatitis A Cases	Cases Reported in Outbreaks		Cases per Transmission Routes					
		Contact		Foodborne		Waterborne					Contact		Foodborne		Waterborne	
		n	% ^1^	n	% ^1^	n	% ^1^	n (%)	n	% ^2^	n	% ^3^	n	% ^3^	n	% ^3^
2002–2006	174 (41.5)	170	97.7	0	0	4	2.3	5524 (40.4)	1252	22.7	1215	97.0	0	0	37	3.0
2007–2011	175 (41.8)	171	97.7	1	0.6	3	1.7	6596 (48.2)	1329	20.1	1305	98.2	14	1.0	10	0.8
2012–2016	56 (13.4)	55	98.2	1	1.8	0	0	1196 (8.7)	363	30.4	361	99.4	2	3.6	0	0
2017–2021	14 (3.3)	14	100	0	0	0	0	363 (2.7)	46	12.7	46	100	0	0	0	0
Total	419 (100)	410	97.8	2	0.5	7	1.7	13,679 (100)	2990	21.9	2927	97.9	16	0.5	47	1.6

^1^ The proportion of outbreaks in the total number of hepatitis A outbreaks in the observed period. ^2^ The proportion of outbreak cases in the total number of hepatitis A cases in the observed period. ^3^ The proportion of outbreak cases in the total number of hepatitis A outbreak cases according to the route of transmission.

## Data Availability

The data that support the findings of this study are available from the corresponding author upon reasonable request.

## Supplementary Materials

The following supporting information can be downloaded at: https://www.mdpi.com/article/10.3390/v15030753/s1. Figure S1: Seasonal distribution of hepatitis A cases (%) in Serbia in the period 2002-2021; Table S1. Proportion (%) of hepatitis A cases by age groups stratified by sex in Serbia (2002–2021); Table S2. Hospitalization status and classification of hepatitis A cases in Vojvodina, 2002–2021; Table S3. Affected population groups and settings of hepatitis A outbreaks in Vojvodina, 2002–2021.
