# Supplementary material for: Epidemiological Transition and Strategies for the Control of Hepatitis A in Serbia
Source: Viruses. 2023 Mar 15;15(3):753. doi: 10.3390/v15030753 (PMC10056894; doi:10.3390/v15030753)
Supplement: Supplementary file 1 [file viruses-15-00753-s001.zip › Table S1, S2, S3.pdf]

Table S1. Proportion (%) of hepatitis A cases by age groups stratified by sex in Serbia (2002-2021)

		Age groups (years)								
Sub-period	Sex	<5	5-9	10-14	15-19	20-29	30-39	40-49	50-59	>60
2002-2006	**Male**	4.6	12.2	14.5	13.2	20.6	16	10.9	5	3
	**Female**	4.5	15.1	14.6	12.2	19.4	16.9	8.9	5.2	3.2
2007-2011	**Male**	2.9	9.2	10.6	13.5	27.1	17.4	11.9	5.3	2.1
	**Female**	4.5	12.4	11.5	11.5	23.3	16.9	11.7	5.4	2.8
2012-2016	**Male**	3.3	10	12	13	20	14	14.3	8.7	4.7
	**Female**	3	11.2	10.8	11	19.6	17.6	12.2	10.6	4
2017-2021	**Male**	1.5	7.1	10.7	6.6	20.5	18.5	16.3	11.7	7.1
	**Female**	2.3	7.6	10.5	8.8	16.4	13.5	14	19.9	7

Table S2. Hospitalization status and classification of hepatitis A cases in Vojvodina, 2002-2021.

Sub-period	Reported cases		Hospitalized cases		Outbreak cases		Number of cases per outbreak			Classification of outbreak cases					
	n	%	n	%[1]	n	%[2]	Min	Max	Mean	Probable		Confirmed		Unclassified[3]	
										n	%	n	%	n	%
2002-2006	1333	49.4	691	51.8	902	67.7	3	114	33	NA[4]	NA[4]	NA[4]	NA[3]	902	100
2007-2011	1038	38.5	437	42.1	606	58.4	3	115	11	369	60.9	237	39.1	0	0
2012-2016	263	9.8	132	50.2	137	52.1	3	78	8	59	43.1	78	56.9	0	0
2017-2021	62	2.3	19	30.6	17	27.4	NA[5]	NA[5]	NA[5]	0	0	17	100	0	0
Total	2696	100	1279	47.4	1662	61.6	3	115	17.3	428	25.8	332	19.9	902	54.3

[1] The proportion of hospitalized patients in the total number of reported hepatitis A cases in each subperiod.

[2] The proportion of outbreak cases in the total number of reported hepatitis A cases in each subperiod.

[3] Case classifications were conducted starting from 2007; EU case definition was applied from 2017.

[4] Not applicable due to different case classification in the period 2002-2006 (hepatitis A was diagnosed based on the exclusion of hepatitis B and hepatitis C).

[5] Not applicable: only one outbreak of hepatitis A was reported (17 cases) in the period 2017-2021.

Table S3. Affected population groups and settings of hepatitis A outbreaks in Vojvodina, 2002-2021.

Sub-period	Reported outbreaks		Outbreak settings or affected population groups									
			General population[1]		Hospital[1]		Schools[1]		Immigrants, refugees[1]		Roma[1]	
	n	%	n	%	n	%	n	%	n	%	n	%
2002-2006	33	41.8	18	54.5	2	6.1	7	21.2	1	3.0	5	15.2
2007-2011	32	40.5	24	75.0	0	0	4	12.5	0	0	4	12.5
2012-2016	13	16.5	12	92.3	0	0	0	0	1	7.7	0	0
2017-2021	1	1.2	1	100	0	0	0	0	0	0	0	0
Total	79	100	55	69.6	2	2.5	11	14.0	2	2.5	9	11.4

[1] The number and share (%) of outbreaks in relation to the total number of hepatitis A outbreaks in the corresponding subperiod.
