# Supplementary figures and images for: Epidemiological Transition and Strategies for the Control of Hepatitis A in Serbia
Source: Viruses. 2023 Mar 15;15(3):753. doi: 10.3390/v15030753 (PMC10056894; doi:10.3390/v15030753)

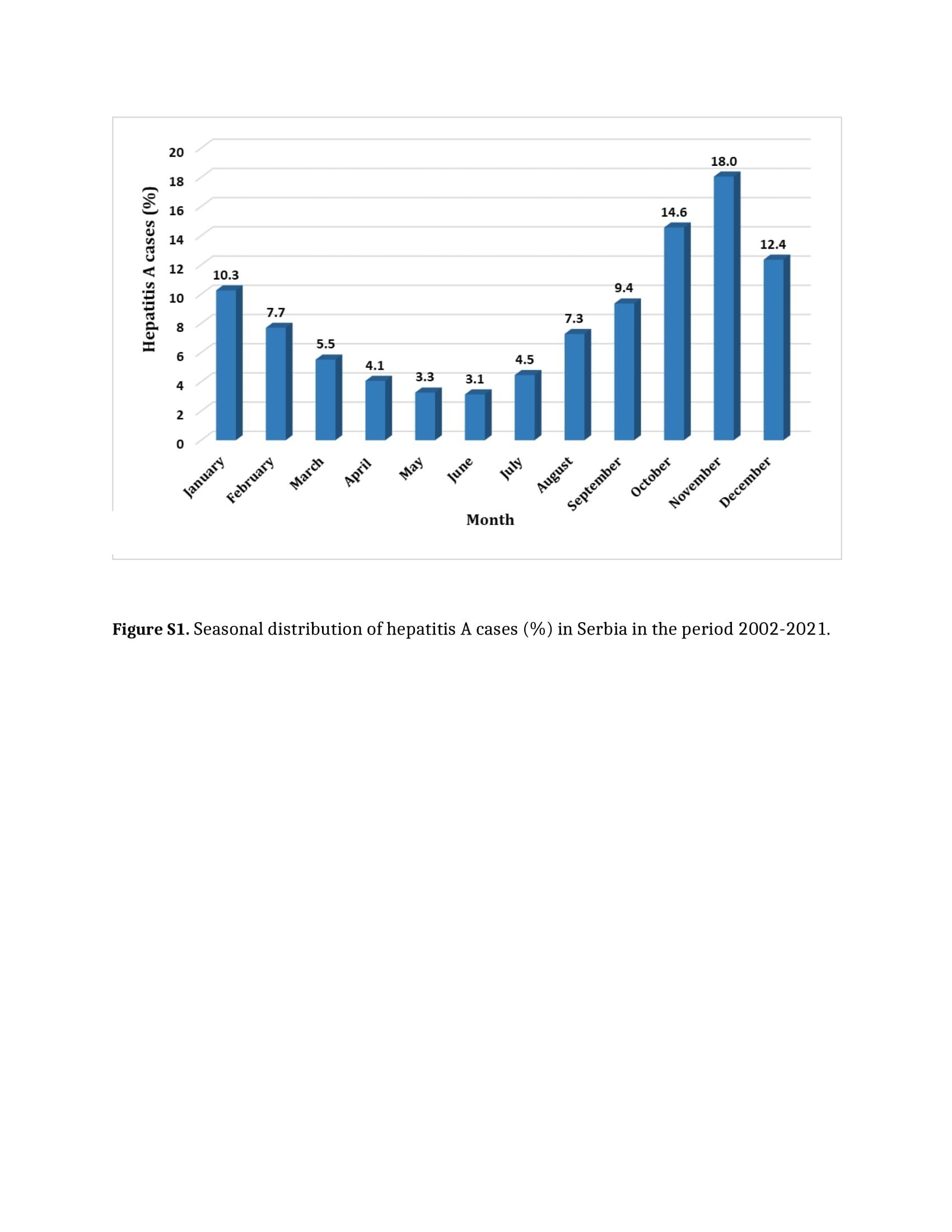

Supplement: Supplementary file 1 [file viruses-15-00753-s001.zip › Figure S1.jpeg]
